# An Integrated Microfluidic Microwave Array Sensor with Machine Learning for Enrichment and Detection of Mixed Biological Solution

**DOI:** 10.3390/bios15010045

**Published:** 2025-01-13

**Authors:** Sen Yang, Yanxiong Wang, Yanfeng Jiang, Tian Qiang

**Affiliations:** 1 School of Integrated Circuits, Jiangnan University, Wuxi 214122, China; 6221922014@stu.jiangnan.edu.cn (S.Y.); 7231923001@stu.jiangnan.edu.cn (Y.W.); jiangyf@jiangnan.edu.cn (Y.J.); 2 School of Internet of Things Engineering, Jiangnan University, Wuxi 214122, China

**Keywords:** microwave array sensor, microwave detection, microfluidic chip, enrichment and detection, mixed biological solution

## Abstract

In this work, an integrated microfluidic microwave array sensor is proposed for the enrichment and detection of mixed biological solution. In individuals with urinary tract infections or intestinal health issues, the levels of white blood cells (WBCs) and *Escherichia coli* (*E. coli*) in urine or intestinal extracts can be significantly elevated compared to normal. The proposed integrated chip, characterized by its low cost, simplicity of operation, fast response, and high accuracy, is designed to detect a mixed solution of WBCs and *E. coli*. The results demonstrate that microfluidics could effectively enrich WBCs with an efficiency of 88.3%. For WBC detection, the resonance frequency of the sensing chip decreases with increasing concentration, while for *E. coli* detection, the capacitance value of the sensing chip increases with elevated concentration. Furthermore, the measurement data are processed using machine learning. Specifically, the WBC measurement data are subjected to a further linear fitting. In addition, the prediction model for *E. coli* concentration, employing four different algorithms, achieves a maximum accuracy of 95.24%. Consequently, the proposed integrated chip can be employed for the clinical diagnosis of WBCs and *E. coli*, providing a novel approach for medical and biological research involving cells and bacteria.

## 1. Introduction

In clinical diagnosis, the concentration of WBCs and *E. coli* is often used as the basis for the diagnosis of related diseases, such as urinary tract infections and intestinal health issues [1,2]. Clinical diagnostic methods such as urinalysis, urine culture, and stool microscopy are comprehensive and accurate, but they are time-consuming, costly, and have lower sensitivity and specificity [3]. However, microwave sensors are widely used to detect various biological samples due to their advantages such as fast response, high sensitivity, simplicity of operation, label-free, ease of integration, multiparameter detection, and non-contact [4,5]. Piekarz proposed a label-free capacitive microwave biosensor detection method, successfully used to detect *E. coli* at various concentrations [6]. Osterberg reported the development of a simple interferometer-based microwave sensing system for multiple frequency characterization and differentiation of in-flow yeast cells [7]. Nonetheless, the previous methods have tested individual samples, but in real scenarios, WBCs and *E. coli* are mixed. Therefore, we propose a method combining microfluidic chip and microwave sensor chip to achieve enrichment and detection of mixed samples.

Microfluidics utilizes microchannels and microcavities to manipulate micro-quantity liquid, enabling precise chemical or biological analysis [8,9,10]. Compared with conventional ultracentrifugation, chemical, and magnetic separation techniques, microfluidics offers superior performance for intact cell detection, low cost, high efficiency, and fast response [11]. Many researchers have demonstrated the feasibility of microfluidic cell enrichment [12,13]. Consequently, an integrated chip combining microfluidics and microwave technology enables the enrichment and detection of complex biological cell samples, overcoming the complexity and high cost of traditional cell detection and clinical diagnostics [14].

With the continuous development of science and technology, artificial intelligence (AI) is widely used in various fields. It can efficiently process and analyze large amounts of complex sensor data to improve the performance and accuracy of sensors [15,16]. Therefore, many researchers have used AI algorithms to analyze the measurement data of microwave sensor arrays to further improve and expand the performance and application of sensors [17,18]. Additionally, many researchers have used microwave sensors combined with microfluidic to achieve accurate detection of liquids [19,20]. However, in the field of biosensing and medical diagnosis, a few researchers have designed a microfluidic and microwave sensor into an integrated chip for cell enrichment and detection, using Machine Learning to analyze and process the measurement results [21]. Aiming to improve the accuracy of microwave sensors, we design the integrated chip and analyze the data using algorithms, then conduct exploratory experiments in the laboratory environment to verify the chip performance.

In this paper, we prepare mixed solution samples of WBCs and *E. coli* for experimental validation of the designed integrated chip in the existing laboratory environment. The measurement results indicate that the integrated chip can enrich and detect mixed samples of WBCs and *E. coli*. Furthermore, Machine Learning algorithms enhance detection accuracy, with multiple linear regression improving WBC linearity by 0.6–6.2%, and prediction models for *E. coli* concentration achieving up to 95.24% accuracy using K-Nearest Neighbor (KNN), Support Vector Machine (SVM), Decision Tree (DT), and Random Forest (RF). Thus, the proposed chip with algorithmic analysis can accurately detect cells and bacteria and is superior to traditional methods in terms of sensitivity, cost, and integration. In summary, the proposed chip holds great potential for integration into existing clinical workflows and for enabling the portability and miniaturization of the device through its combination with a miniature VNA. This work provides a more efficient and convenient method for medical diagnosis of WBCs and *E. coli*, and offers a reliable approach for medical and biological research on cells and bacteria.

## 2. Materials and Methods

### 2.1. Design and Validation of Microwave Array Sensor

**Figure 1:**
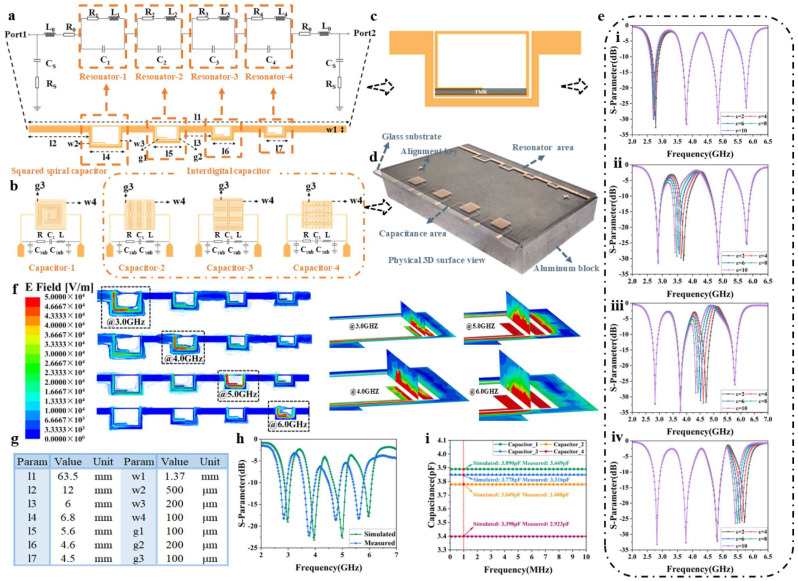
The proposed microwave sensor based on the glass-based IC process: (**a**) Equivalent circuit and structure of microwave resonator array; (**b**) Structure and equivalent circuit of capacitor array; (**c**) The position of the MTR on the resonator; (**d**) Physical image of the fabricated sensor; (**e**) The result of S_21_ is when MTR is on (**e**-**i**) Resonator-1, (**e**-**ii**) Resonator-2, (**e**-**iii**) Resonator-3, and (**e**-**iv**) Resonator-4; (**f**) Electric field distribution at resonance frequency in the horizontal plane; (**g**) Dimensional table of sensor; (**h**) ADS Simulated and measured results of microwave resonator array; (**i**) Simulated and measured capacitance values of capacitor array at 1 MHz.

In this study, the sensing chip is divided into two components, a microwave resonator array for measuring the enriched liquid at the microfluidic Outlet-1, and a capacitor array for measuring the enriched liquid at the microfluidic Outlet-2. Initially, the microwave resonator array, consisting of four square helical resonators, is designed using Advanced Design System (ADS 2022) and High Frequency Structure Simulator (HFSS AnsysEM19.1) software. The substrate is 1 mm thick glass with an effective dielectric constant of 6.7 and a loss tangent of 0.0059. The metallic part of the sensor is composed of copper with a thickness of 500 nm. The resonance frequency is determined by the following formula:

(1) $$f=\frac{1}{2\pi \sqrt{L_{\mathrm{out}}\left(C_{g}+C_{s}\right)}}$$

where $L_{out}$ represents the inductance effect generated by the metals at high frequencies, $C_{g}$ is the inter-electrode capacitance of the metal line gaps, and $C_{s}$ is the capacitance between substrates. The value of $C_{s}$ is very small compared to $C_{g}$, so its effect on the resonance frequency is negligible. Figure 1a illustrates the equivalent circuit diagram and specific structure of the sensing resonator array. The equivalent inductance in the resonant circuit can be approximated by the following formula:

(2) $$L_{\mathrm{out}}=\mu_{0}\mu_{r}\frac{\mathrm{LD}}{H}$$

where *H* and *D* represent the intensity and depth of the magnetic field in the open-loop resonator, *L* is the length of one side of the square ring, $\mu_{0}$ is the vacuum permeability, and $\mu_{r}$ is the relative permeability. Similarly, the equivalent capacitance formed by the electric field between the metal lines can be obtained:

(3) $$C_{g}=\mu_{0}\mu_{r}\frac{A}{d}$$

where *A* is the area of the square ring capacitance region and *d* is the gap width of the ring. Furthermore, the capacitor array in Figure 1b consists of a square spiral capacitor and three interdigital capacitors, constructed with the same materials and thickness as the microwave resonator array. Additionally, these four capacitors have a line width and spacing of 0.1 mm and operate at a frequency of 1 MHz with capacitance values of 3.449 pF, 3.316 pF, 3.488 pF, and 2.923 pF. Correspondingly, their equivalent circuit diagrams are RLC series circuits, calculated according to the following equations. For the square spiral capacitor:

(4) $$C_{s}=\frac{\varepsilon_{0}\varepsilon_{r}A}{d}$$

where $\varepsilon_{r}$ is the relative permittivity of the substrate material, $\varepsilon_{0}$ is the permittivity of free space, A is the effective area of the electrodes, and d is the distance between the electrodes. For the interdigital capacitors, the equivalent capacitance is calculated as follows:

(5) $$C_{s}=\frac{\varepsilon_{0}\varepsilon_{r}\mathrm{WL}(N-1)}{d}$$

where N is the number of interdigital electrodes, W is the electrode width, L is the electrode length, and d is the electrode spacing.

Finally, the entire sensing chip consists of a microwave resonator array and a capacitor array together, as shown in Figure 1d. Figure 1e illustrates the S_21_ results for TMR at different resonators in Figure 1c. Figure 1f illustrates the electric field distribution of the microwave resonator array, demonstrating the strong electric field strength and sensitivity of the designed device. The exact dimensions of this sensor are shown in Figure 1g. The ADS simulated and measured S_21_ parameters of the resonator array are shown in Figure 1h, while the simulated and measured values of the capacitor array at 1 MHz are presented in Figure 1i. The close proximity of the measured values to the simulated values in the above results confirms that both the design and the fabrication of the device are highly feasible.

### 2.2. Design and Validation of Microfluidic Chip

**Figure 2:**
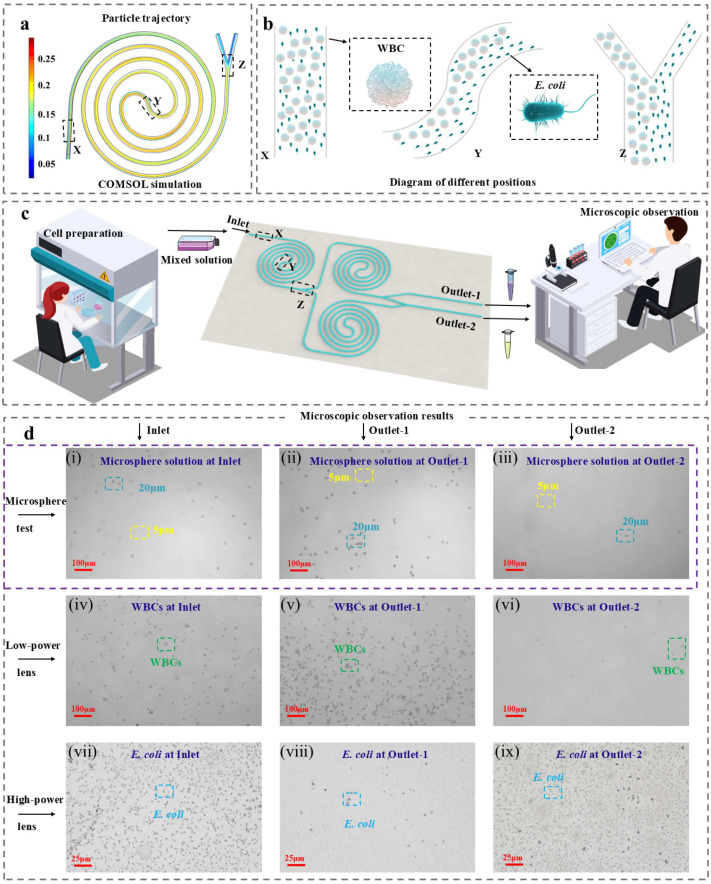
Design and verification of a microfluidic chip: (**a**) Particle trajectories of 20 µm and 5 µm simulated by COMSOL Multiphysics; (**b**) Focusing of WBCs and *E. coli* in different locations; (**c**) Microfluidic sorting process and structure diagram; (**d**) Microfluidic verification results observed by microscopy: (**d**-**i**) Mixed solutions of microspheres at Inlet, (**d**-**ii**) Outlet-1, and (**d**-**iii**) Outlet-2; (**d**-**iv**) WBCs under low magnification microscope at Inlet, (**d**-**v**) Outlet-1, and (**d**-**vi**) Outlet-2; (**d**-**vii**) *E. coli* under high magnification at Inlet, (**d**-**viii**) Outlet-1, and (**d**-**ix**) Outlet-2.

The inertial approach has been applied to blood separation for various biomedical research studies [22]. Particularly, spiral channels offer a simple and efficient solution to achieve sustainable separation based on particle size without the need for complex microstructures or additional components. The microfluidic in this paper is designed through COMSOL Multiphysics software (6.0) simulation with a channel width of 200 μm and a height of 100 μm, the simulation focuses on the physical fields of laminar flow and particle tracking, incorporating two force conditions, i.e., influenced by inertial lift forces (*F_L_*) and viscous drag forces (*F_D_*), to model the fluid dynamics within the microfluidic channel. Figure 2a presents the simulation result of particle paths in the designed single spiral microfluidic chip, while Figure 2b illustrates particle focusing at different positions. As the fluid enters the spiral microfluidic chip, suspended particles in the fluid slowly move along the channel, gradually influenced by inertial lift forces and viscous drag forces. Inertial lift forces include shear-induced lift forces due to the parabolic velocity distribution within the finite channel and wall-induced lift forces generated by disturbance-induced swirling flow near the wall [23]. For particles with $a_{p}/D_{h}\geq \;$0.07 [24], the interaction between shear-induced lift and wall-induced lift forces causes particles initially randomly distributed to migrate laterally to stable equilibrium positions around the periphery of the microchannel. Here, $a_{p}$ denotes particle diameter and $D_{h}=4\;A/P$ represents the hydraulic diameter of the microfluidic chip, where A and P, respectively, denote the cross-sectional area and perimeter of the microfluidic chip. The magnitude of inertial lift forces depends on both particle size and fluid shear flow velocity, as expressed in the following equation:

(6) $$F_{L}=k\rho U_{m}^{2}a_{p}^{3}$$

where k is a dimensionless coefficient that depends on the position of the particle and the cross-sectional shape of the pipe, ρ is the density of the fluid, and $U_{m}$ is the average velocity of the fluid [25]. However, secondary flows consisting of two counter-rotating Dean Vortices occur in the cross-section of the spiral microfluidic chip due to the centrifugal acceleration of the fluid [26]. The intensity of these vortices can be represented by the Dean Drag Force:

(7) $$F_{D}=\frac{3.96\rho U_{m}^{2}a^{2}D_{h}}{R_{c}}$$

where $R_{c}$ is the radius of curvature of the spiral channel. In spiral microchannels, the combined effects of Dean Resistance and inertial lift forces reduce particles to two equilibrium positions, enabling size-based particle enrichment by causing different diameters to occupy distinct lateral positions near the inner wall.

Two experiments are conducted to test the performance of the microfluidic chip: one to verify the enrichment capability with a mixed microsphere solution containing 20 µm and 5 µm particles, and the other to test with a mixed solution of WBCs (10–15 µm) and *E. coli* (<1 µm). Next, the mixed solution is introduced into the microfluidic chip, as depicted in Figure 2c. Finally, the samples collected from two outlets are observed under a microscope, and the observation results are presented in Figure 2d. Based on particle size, WBCs and microspheres are observed under 5× magnification, while *E. coli* requires 20× magnification for visibility. The results show that most of the large particles are concentrated in Outlet-1, while the small particles tend to concentrate in Outlet-2 and are partially filtered out, resulting in achieving the enrichment of large particles in Outlet-1. In summary, the microfluidic chip demonstrates excellent enrichment performance on both actual cells and microspheres. The results indicate the potential and feasibility of using the designed microfluidic chip for enriching other biological samples or human body fluid extracts. Additionally, it shows the potential to apply the separation of microplastics.

### 2.3. Preparation of Biological Samples

The THP-1 human blood monocyte cell line is sourced from the Cell Bank of the Chinese Academy of Sciences, Shanghai. The cells are cultured in RPMI 1640 medium (Biological Industries, Beit Haemek, Israel) supplemented with 10% fetal bovine serum (Biological Industries, Beit Haemek, Israel) and 1% penicillin-streptomycin (Biological Industries, Beit Haemek, Israel). Cultivation occurs under conditions of 37 °C with 5% CO_2_ in a humidified cell culture incubator (Thermo-Fisher, Waltham, MA, USA). Routine maintenance involves the passage of THP-1 cells into cell culture flasks (Corning, New York, NY, USA) every two days. Harvesting is achieved by centrifugation at 1400 rpm for 4 min using 50 mL centrifuge tubes. Following centrifugation, the supernatant is aspirated, and cells are resuspended in fresh culture medium to a concentration of 10^4^/mL before reintroduction into the incubator.

The DH5α strain of *Escherichia coli* (*E. coli*) used in this experiment is obtained from the Microbial Culture Collection at the Key Laboratory of Carbohydrate Chemistry and Biotechnology (Jiangnan University), Ministry of Education, China. Bacteria are cultured in Lysogeny Broth (LB) medium (dissolve 10 g of tryptone, 5 g of yeast extract, and 10 g of NaCl in 1 L of distilled water, adjust the pH of the solution to 7.0, and autoclave at 115 °C under high pressure for 15 min to sterilize—reagents are all from Sangon, Shanghai, China). For cultivation, 1 μL of bacteria from the culture collection is inoculated into a test tube containing 3 mL of LB medium. The tube is then placed into a constant-temperature shaking incubator set at 37 °C and 220 rpm for 12 h. Upon completion of the incubation period, bacterial cells are harvested by centrifugation at 8000 rpm for 5 min. The resulting pellet is resuspended thoroughly in PBS buffer containing 1% BSA, and subsequently, diluted to the desired concentration for further use. The images of the detailed cell preparation process are shown in Figure S1 in Supplementary Note S1.

Each batch of cells is cultured for 4–7 days, and the growth state of the cells is observed under a microscope. Once the cell status and quantity meet the experimental requirements, the cells are placed in a centrifuge, to extract the cell samples. After extraction, the cell samples are prepared in a sterile workbench. Initially, the cell number is recorded using the cell and bacterial counting instruments. The white blood cells (WBCs) are counted by cell counting plates (Thermo-Fisher, Waltham, MA, USA) and a cell counter (Thermo-Fisher, Waltham, MA, USA), while the optical density (OD) of *E. coli* is measured using a spectrophotometer (Ultrospec 2100 pro, Harvard Bioscience, Inc., Holliston, MA, USA) at 600 nm wavelength. The concentration is then calculated using the following formula:

(8) $$Y=5.904\times 10^{7}\cdot \mathrm{OD}+1.085\times 10^{6}$$

**Figure 3:**
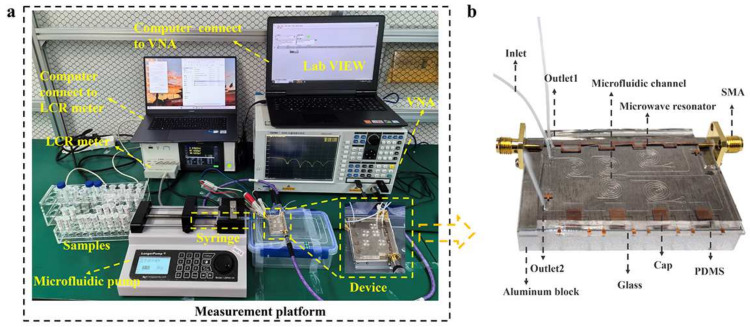
Experimental environment and devices: (**a**) Measurement platform for experiments; (**b**) Integrated chip physical drawing.

The solution of the required specific concentration is configured according to the initial concentration measured by the counting instruments. The concentration of WBCs in human blood typically ranges from 4000 to 11,000 cells per microliter, which corresponds to 4 × 10^6^/mL to 1.1 × 10^7^/mL. The concentration of *E. coli* in the human body, particularly in the intestines, typically ranges from 10^6^ to 10^9^ bacteria per gram of feces. In the human body, mixed solutions of WBCs and *E. coli* can be obtained from locations such as the intestinal tract and urine. The concentrations in these samples are typically lower than normal physiological levels. Therefore, the order of magnitude for preparing mixed solutions of WBCs and *E. coli* is chosen to be 10^5^/mL. The biological samples are then subjected to experimental validation using the measurement platform depicted in Figure 3a.

### 2.4. Integrated Chip Manufacturing Process

The integrated chip proposed in this paper is initially fabricated, resulting in a standard-sized aluminum block, a microwave sensor evaporated with copper on a glass substrate, and a PDMS microfluidic chip. First, a fixing point for the SMA connector is drilled into the aluminum block based on the position of the transmission line. Next, PDMS prepolymer and crosslinker are mixed thoroughly in a 10:1 ratio and then spread evenly onto the mask. This mixture is degassed in a vacuum desiccator for 2 h to remove any air bubbles. It is then cured at 85 °C for 90 min. Finally, the PDMS is allowed to sit for 1–2 h prior to cutting and drilling. PDMS and glass substrate are processed through oxygen plasma surface activation for 90 s. Then, the aligned surfaces are combined and heated in a vacuum desiccator at 65 °C for 30 min. After standing for 1–2 h, adhesive components can be used. This paper has solved most of the challenges in the manufacturing process by mastering the aforementioned process independently, resulting in very low overall manufacturing costs. The SMA attaches the aluminum block to the adhesive chip, as shown in Figure 3b.

## 3. Results and Discussion

### 3.1. White Blood Cell Enrichment Results

**Figure 4:**
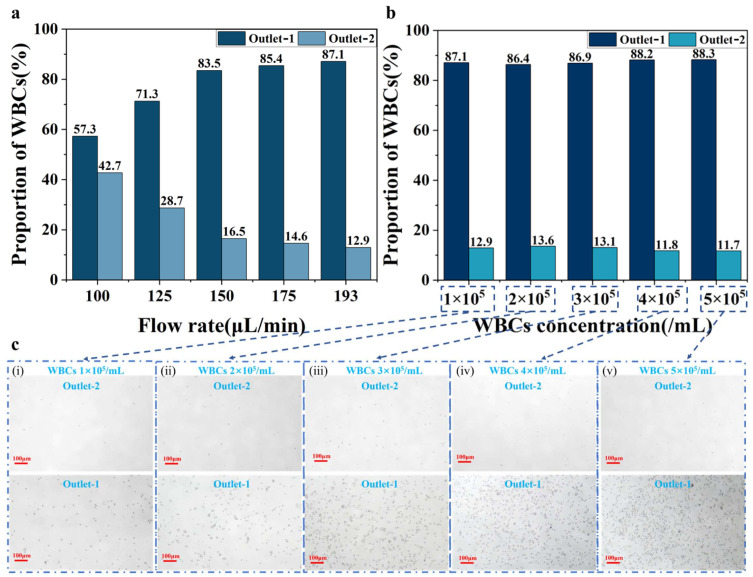
WBC enrichment results: (**a**) Enrichment effect of WBCs at different flow rates; (**b**) WBC enrichment results for different concentrations; (**c**) Microscopic observation of WBC enrichment at different concentrations, observation of WBC concentration of (**c**-**i**) 1 × 10^5^/mL, (**c**-**ii**) 2 × 10^5^/mL, (**c**-**iii**) 3 × 10^5^/mL, (**c**-**iv**) 4 × 10^5^/mL, and (**c**-**v**) 5 × 10^5^/mL.

When the microfluidic chip is fixed, the flow rate is the main factor affecting the enriching effect. Previously, the optimal flow rate of 180 μL/min is obtained using COMSOL Multiphysics. Since the flow rate adjustment range of the microfluidic pump is 0–193 μL/min, therefore, the enrichment efficiency of WBCs with concentration of 1 × 10^5^/mL is investigated within the flow rate range of 100–193 μL/min. The experiments are repeated three times for each flow rate to obtain the average result, as shown in Figure 4a. It is clear that the efficiency of WBC enrichment increases with higher flow rates. The reason for this phenomenon is that for a certain range of flow rates, the inertial lift forces and viscous drag forces on the particles are enhanced with increasing flow rate, which helps the particles to reach the equilibrium position faster. However, if the flow rate is too low, the particles cannot achieve proper focus, while an excessively high flow rate increases the interaction forces between particles. Both situations reduce the enrichment efficiency of the WBCs. Therefore, the optimal flow rate for microfluidic enrichment in this paper is selected as 193 μL/min.

Subsequently, mixed solutions of five different concentrations of WBCs (1 × 10^5^–5 × 10^5^/mL) and one concentration of *E. coli* (1 × 10^5^/mL) are prepared. The different concentrations of Outlet-1 fluid obtained at 193 μL/min are subjected to cell counting and microscopic observation. As shown in Figure 4b, the results indicate WBC enriching efficiencies of 87.1%, 86.4%, 86.9%, 88.2%, and 88.3% from low to high concentrations respectively, proving the microfluidic enrichment effect on WBCs. Additionally, glass slides are observed under a microscope to examine the enriching effect at the five concentrations as shown in Figure 4c. WBCs in Outlet-1 are significantly more abundant than in Outlet-2, and the WBC concentration in Outlet-1 increased with higher concentrations, consistent with the cell counting results in Figure 4b. In summary, WBCs are focused toward Outlet-1 under the effect of inertial lift forces and viscous drag forces at a flow rate of 193 μL/min. The results demonstrate that the designed microfluidic could be applied to the enrichment of biological cells.

### 3.2. Microwave Sensing Response Results

**Figure 5:**
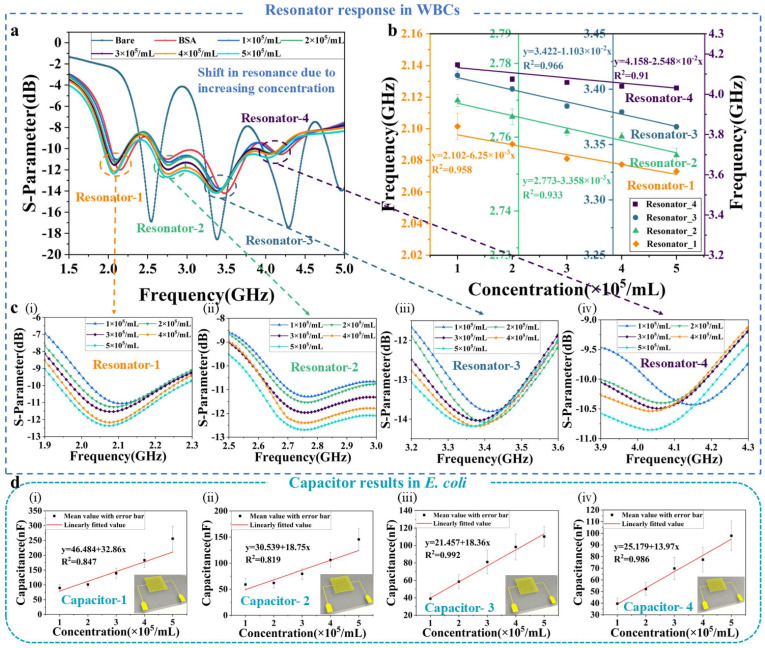
Microwave sensor detection results for WBCs and *E. coli*: (**a**) S-parameters of microwave resonator array for different concentrations of WBCs; (**b**) Linearly fitted diagrams including the mean value of resonance frequency with error bars corresponding to WBCs; (**c**) Detailed diagram of resonator’s frequency shift in WBC concentration: (**c**-**i**) Resonator-1, (**c**-**ii**) Resonator-2, (**c**-**iii**) Resonator-3, and (**c**-**iv**) Resonator-4; (**d**) Capacitance measurements of capacitor array for different concentrations of *E. coli*, fitted line diagrams including the mean value of capacitor’s frequency with error bars corresponding to *E. coli* at (**d**-**i**) Capacitor-1, (**d**-**ii**) Capacitor-2, (**d**-**iii**) Capacitor-3, and (**d**-**iv**) Capacitor-4. Note: Error bars are generated by fitting multiple measurement data using standard deviation (SD < 3.5%).

In the preliminary experiment, an interdigitated capacitor is used at a frequency of 1 MHz. A pipette is used to sequentially extract 20 μL of WBC solutions with concentrations of 1 × 10^5^/mL, 2 × 10^5^/mL, 3 × 10^5^/mL, 4 × 10^5^/mL, and 5 × 10^5^/mL, which are dispensed onto the sensitive area of the capacitor, fully covering it. Each concentration is measured three times, and the average value is calculated to ensure data accuracy. The results indicate that the capacitance values increased with higher concentrations, which are 76 nF, 105 nF, 139 nF, 165 nF, and 198 nF. The experiment demonstrates that the dielectric constant of the solution changes with increasing cell concentration, leading to an increase in the capacitance value of the microwave device structure. This change in the dielectric constant is detected by the microwave sensor. The microwave resonator response at Outlet-1 with different concentrations of mixed solutions is shown in Figure 5a, which indicates that the resonance frequency decreases with the increasing concentration of WBCs. The cause of this response is the effective dielectric constant of WBCs, which increases with the elevated concentration, leading to a rise in the capacitance value of the resonator structure, and ultimately leading to a decrease in the resonance frequency. To minimize experimental tolerance, each concentration gradient is measured three times.

Figure 5b presents the linear fit curves of the measurement results for the four resonators. Figure 5c displays the microwave responses of the four resonators with varying concentrations. The absolute sensitivities of the four resonators in the sensor array for detecting WBC concentration changes are 6.25 MHz/10^5^·mL^−1^, 3.358 MHz/10^5^·mL^−1^, 11.03 MHz/10^5^·mL^−1^, and 25.48 MHz/10^5^·mL^−1^, respectively. The designed microwave sensor exhibits high sensitivity to changes in WBC concentration, since the higher concentration of WBCs obtained by microfluidic enrichment makes the response of the microwave device more sensitive. Similarly, the sensitivity of the capacitor array to different concentrations of *E. coli* at Outlet-2 is obtained by experiments with mixed solutions of five concentrations of *E. coli* (1 × 10^5^–5 × 10^5^/mL) and one concentration of WBCs (1 × 10^5^/mL). The results of the four capacitor sensors with varying concentrations of *E. coli* are shown in Figure 5d. As the concentration of *E. coli* elevates, the effective dielectric constant of the solution increases, resulting in a higher capacitance value. The sensitivities of the four capacitor sensors are 32.86 nF/10^5^·mL^−1^, 18.75 nF/10^5^·mL^−1^, 18.36 nF/10^5^·mL^−1^, and 13.97 nF/10^5^·mL^−1^, respectively. Detailed statistical parameters can be used to further verify the performance of the sensors, as shown in Table S1 in Supplementary Note S2. In summary, the integrated chip can detect a mixed solution of WBCs and *E. coli* quickly and accurately.

### 3.3. Machine Learning Algorithm Analysis

**Figure 6:**
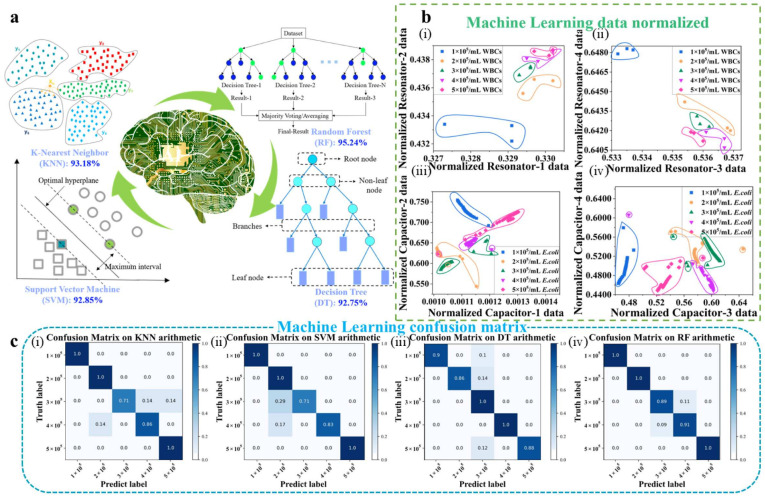
Machine Learning data processing: (**a**) Machine Learning algorithms analyze the experimental data; (**b**) Normalized processing of data distribution maps drawn by (**b**-**i**) Resonator-1 and Resonator-2, (**b**-**ii**) Resonator-3 and Resonator-4, (**b**-**iii**) Capacitor-1 and Capacitor-2, and (**b**-**iv**) Capacitor-3 and Capacitor-4; (**c**) Confusion matrix of, (**c**-**i**) KNN, (**c**-**ii**) SVM, (**c**-**iii**) DT, and (**c**-**iv**) RF.

Traditional microwave sensors are primarily dependent on hardware for data acquisition and processing, which could be limited in the handling of complex and large-scale data. However, AI can significantly enhance the ability of microwave sensors to process data. With the introduction of AI technology, sensors can analyze large amounts of data more efficiently, improving data accuracy and reliability [27]. Machine Learning has been proven to enhance datasets accuracy in aiding microwave sensors [28,29]. Algorithms such as SVM, KNN, DT, and RF have shown promising results in dealing with the predictive analysis and classification of microwave sensor measurement datasets [30]. Therefore, processing by Machine Learning algorithms can further reduce the impact of bias and improve the accuracy of the datasets, as illustrated in Figure 6a.

The sensor design includes four resonators and capacitors, with frequency used as a feature for WBCs (15 samples, 80% training, 20% testing) and capacitance used as a feature for *E. coli* (140 samples, 70% training, 30% testing). Since the cost of culturing *E. coli* is much lower than that of WBCs, and microwave measurements are more complex than capacitance measurements, more *E. coli* data are obtained than WBCs. Therefore, multiple linear fitting algorithm is used to analyze the measurements of the WBCs, and other algorithms are used to analyze the measurements of the *E. coli*. All data are normalized to make targets comparable across different features without changing the distribution of feature data, as shown in Figure 6b. Normalization in Machine Learning can scale feature data to the same range or to a mean of 0 and a variance of 1, and further prevent overfitting by cross-validation in the training set, improving model training convergence speed and performance.

Firstly, the goodness of fit of individual resonators for WBCs is over 90% and the sample data are relatively small, making it difficult for some classification algorithms to delineate class boundaries. Therefore, multiple linear fitting algorithms can be used for data processing. Compared to single resonator’s frequency fitting, four resonator’s frequency linear fitting has higher accuracy and fit. If deviations occur in one resonator feature, the other three resonator features can effectively correct these deviations. A multivariate linear fit is performed on the unnormalized data and the results are shown below:

*y =* 34,750,985 − 1,782,768.2*x*_1_ − 3,790,060*x*_2_ − 6,226,079.5*x*_3_ − 204,839.83*x*_4_ *R*^2^ = 0.9542 (9)

Compared to the single resonator feature fit in Figure 5b, the data-normalized multivariate linear fitting model has a goodness of fit of 97.27%, which is an improvement of 1.47%, 3.97%, 0.6%, and 6.27%, respectively, as follows:

*y* = 6.272601 × 10^9^ − 2.0640911 × 10^9^*x*_1_ − 2.6435233 × 10^9^*x*_2_ − 3.4224074 × 10^9^*x*_3_ − 4.0489418 × 10^9^*x*_4_ *R*^2^ = 0.9727 (10)

Secondly, *E. coli* data have clear class boundaries and large sample numbers, and therefore, the performance of four popular classifiers, SVM, KNN, DT, and RF, are compared. Correspondingly, the details of each method are as follows: for SVM, a polynomial kernel function and a regularization coefficient of 1.0 are used; for KNN, k = 3 means 3 neighbors; for DT, the tree depth is set to 4, with a random seed of 42; and for RF, 20 DTs are used. The model accuracies obtained before data standardization are 88.67%, 90.47%, 76.19%, and 78.57%, respectively. After normalization, the model accuracies are 93.18%, 92.85%, 92.75%, and 95.24%, respectively. These model accuracies represent significant improvements compared to other microwave sensor measurement datasets processed using Machine Learning [31].

In addition, the confusion matrix is used in Machine Learning to evaluate the performance of a classification model. The confusion matrix after training of the four models in this paper is given in Figure 6c, indicating that all four models have good performance. To summarize, all five algorithms perform well on the experimentally obtained datasets, which can further improve the stability and accuracy of the sensors when used in real-world scenarios. Therefore, the designed sensors have the potential to be used in complex environments and to detect complex samples. The performance comparison with other works is shown in Table 1. The proposed sensor has higher enrichment efficiency, more sensing units, higher sensitivity, and algorithm accuracy.

### 3.4. Sensing Mechanism Analysis and Overall Experimental Process

**Figure 7:**
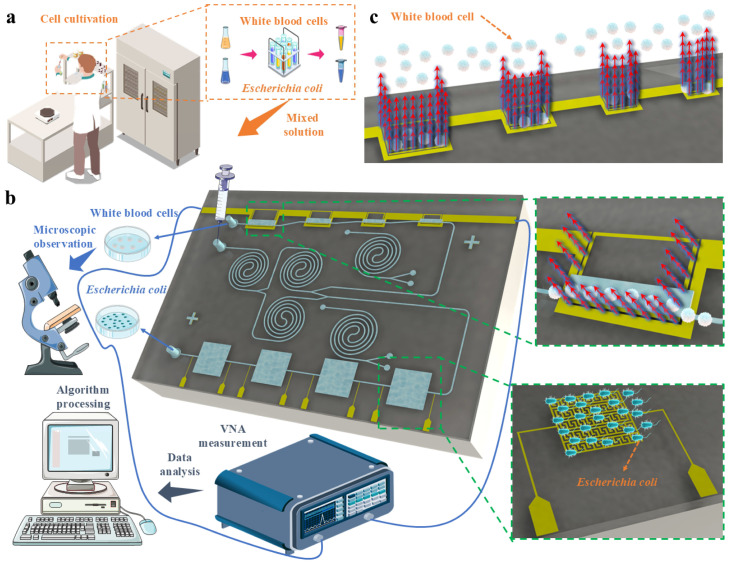
Sensing mechanism and experimental process: (**a**) Preparation of WBCs and *E. coli* samples; (**b**) Schematic diagram of integrated chip operation and sensing mechanism; (**c**) Schematic diagram of WBC sensing mechanism.

The fundamental principle behind the detection of WBC concentration using a microwave resonator is the interaction between electromagnetic waves and biological samples. The microwave resonator generates resonance through a microwave electromagnetic field. When the resonator interacts with the WBC sample to be detected, the electromagnetic properties of the WBCs affect the resonance frequency and quality factor, as shown in Figure 7b,c. The primary electromagnetic properties of the WBC sample are the dielectric constant and conductivity. The dielectric constant varies with different concentrations of WBCs, thus impacting the resonance frequency differently. The effective dielectric constant is calculated by following formula:

(11) $$\varepsilon_{\mathrm{eff}}=\varepsilon_{m}(\frac{\varepsilon_{i}+2\varepsilon_{m}+2f(\varepsilon_{i}-\varepsilon_{m})}{\varepsilon_{i}+2\varepsilon_{m}\;-f(\varepsilon_{i}-\varepsilon_{m})})$$

where $\varepsilon_{eff}$ is the effective dielectric constant, $\varepsilon_{m}$ is the dielectric constant of the matrix (PBS + 1% BSA solution in this paper), $\varepsilon_{i}$ is the dielectric constant of the WBCs, and f is the volume fraction of the WBCs. As the concentration of WBCs increases, their volume fraction increases, leading to an increase in the effective dielectric constant, thus affecting the resonance frequency.

The capacitor detects changes in *E. coli* concentration by measuring the dielectric constant and conductivity of the *E. coli* sample, as shown in Figure 6b. When the concentration of *E. coli* rises, its dielectric constant also increases. The effective dielectric constant is calculated similarly to the aforementioned formula. Figure 7 illustrates the overall methodology and basic sensing principles of this paper.

## 4. Conclusions

In this paper, an integrated microfluidic microwave biosensing chip is designed to enrich and detect mixed solutions of WBCs and *E. coli*. The datasets are analyzed and processed by AI algorithms. The proposed detection method features simplicity of operation, low cost, high sensitivity, and accuracy. It quickly and accurately detects the concentration of WBCs and *E. coli*. The method has potential applications in the medical diagnosis of urinary tract infections and intestinal problems, providing a new avenue for biological research and medical diagnosis.

## Figures and Tables

**Table 1:** Performance comparison to other previously reported analogous methods.

Ref.	Microfluidic Structure	Maximum Enrichment Efficiency	Number of Sensors	Maximum Sensitivity	Number of Algorithms	Maximum Accuracy
[19]	A single channel is used only for sensitive areas	None	1	Not Given	2	Not Given
[30]	A single channel is used only for sensitive areas	None	1	Not Given	2	Not Given
[31]	None	None	4	Not Given	3	83.00%
[32]	Single-machine spiral microfluidic	85%	None	None	None	None
[33]	None	None	1	Not Given	1	90.70%
This work	Multistage inertial spiral microfluidics	88.30%	8	25.48 MHz/10^5^·mL^−1^	5	97.27%

## Data Availability

Data are contained within the article and Supplementary Materials.

## Supplementary Materials

The following supporting information can be downloaded at: https://www.mdpi.com/article/10.3390/bios15010045/s1, Figure S1: Preparation of WBCs and *E. coli* samples; Table S1: Statistical analysis of calculation results. References [34,35,36,37,38] are cited in the Supplementary Materials.

## References

[1] Klein R.D., Hultgren S.J. (2020). Urinary tract infections: Microbial pathogenesis, host-pathogen interactions and new treatment strategies. Nat. Rev. Microbiol..

[2] Zhang Y., Bai J.H., Wu H., Ying J.Y. (2015). Trapping cells in paper for white blood cell count. Biosens. Bioelectron..

[3] Grey B., Upton M., Joshi L.T. (2023). Urinary tract infections: A review of the current diagnostics landscape. J. Med. Microbiol..

[4] Jain M.C., Nadaraja A.V., Mohammadi S., Vizcaino B.M., Zarifi M.H. (2021). Passive microwave biosensor for real-time monitoring of subsurface bacterial growth. IEEE Trans. Biomed. Circuits Syst..

[5] Lei Y.J., Zhang D.J., Wang Q.J., Mao S., Kim E.S., Kim N.Y., Zhou Q.H., Li Y.Y., Yao Z. (2025). Detection of carcinoembryonic antigen specificity using microwave biosensor with machine learning. Biosens. Bioelectron..

[6] Piekarz I., Gorska S., Odrobina S., Drab M., Wincza K., Gamian A., Gruszczynski S. (2020). A microwave matrix sensor for multipoint label-free *Escherichia coli* detection. Biosens. Bioelectron..

[7] Osterberg J.A., Dahal N., Divan R., Miller C.S., Moline D., Caldwell T.P., Yu X., Harcum S.W., Wang P. (2021). Microwave sensing of yeast cell species and viability. IEEE Trans. Microw. Theory Tech..

[8] Battat S., Weitz D.A., Whitesides G.M. (2022). An outlook on microfluidics: The promise and the challenge. Lab Chip.

[9] Yeo L.Y., Chang H.C., Chan P.P.Y., Friend J.R. (2010). Microfluidic devices for bioapplications. Small.

[10] Wang X., Xu Y.D., Liu J.L., Zhang Q.J., Yin H.Y., Zhang C., Belfiore L.A., Mao S., Tang J.G. (2025). Control of photothermal liquid jets through microbubble regulation: Fundamental mechanisms and developing strategies. Opt. Laser Technol..

[11] Nge P.N., Rogers C.I., Woolley A.T. (2013). Advances in microfluidic materials, functions, integration, and applications. Chem. Rev..

[12] Yang Y.Y., Noviana E., Nguyen M.P., Geiss B.J., Dandy D.S., Henry C.S. (2017). Paper-based microfluidic devices: Emerging themes and applications. Anal. Chem..

[13] Xiang N., Ni Z.H. (2022). High-throughput concentration of rare malignant tumor cells from large-volume effusions by multistage inertial microfluidics. Lab Chip.

[14] Dai L., Zhao X., Guo J.C., Feng S.L., Fu Y.S., Kang Y.J., Guo J.H. (2020). Microfluidics-based microwave sensor. Sens. Actuators A Phys..

[15] Sun T.M., Feng B., Huo J.P., Xiao Y., Wang W.G., Peng J., Li Z.H., Du C.J., Wang W.X., Zou G.S. (2024). Artificial intelligence meets flexible sensors: Emerging smart flexible sensing systems driven by machine learning and artificial synapses. Nano-Micro Lett..

[16] Moradkhani A., Hasannejad O., Baghelani M. (2022). An artificial intelligence assisted distance variation robust microwave sensor for biofuel analysis applications. IEEE Microw. Wirel. Compon. Lett..

[17] Niksan O., Colegrave K., Zarifi M.H. (2023). Battery-free, artificial neural network-assisted microwave resonator array for ice detection. IEEE Trans. Microw. Theory Tech..

[18] Wu J.K., Yue W., Gao K., Von Gratowski S., Gu X.F., Pan L.J., Kim N.Y., Liang J.G. (2023). Microwave-sensor array for decoupling detection of distance, shape, dielectric, and morphology. IEEE Trans. Instrum. Meas..

[19] Loutchanwoot P., Harnsoongnoen S. (2022). Microwave microfluidic sensor for detection of high equol concentrations in aqueous solution. IEEE Trans. Biomed. Circuits Syst..

[20] Ye W., Wang D.W., Wang J., Chen S.C., Wang G.F., Zhao W.S. (2021). Microwave heating induced on-demand droplet generation in microfluidic systems. Anal. Chem..

[21] Antonelli G., Filippi J., D’Orazio M., Curci G., Casti P., Mencattini A., Martinelli E. (2024). Integrating machine learning and biosensors in microfluidic devices: A review. Biosens. Bioelectron..

[22] Wu Z.L., Chen Y., Wang M.R., Chung A.J. (2016). Continuous inertial microparticle and blood cell separation in straight channels with local microstructures. Lab Chip.

[23] Petchakup C., Chen Y.Y.C., Tay H.M., Ong H.B., Hon P.Y., De P.P., Yeo T.W., Li K.H.H., Vasoo S., Hou H.W. (2023). Rapid screening of urinary tract infection using microfluidic inertial-impedance cytometry. ACS Sens..

[24] Ying Y., Lin Y. (2019). Inertial focusing and separation of particles in similar curved channels. Sci. Rep..

[25] Bhagat A.A.S., Kuntaegowdanahalli S.S., Papautsky I. (2008). Inertial microfluidics for continuous particle filtration and extraction. Microfluid. Nanofluidics.

[26] Wu L.D., Guan G.F., Hou H.W., Bhagat A.A.S., Han J. (2012). Separation of leukocytes from blood using spiral channel with trapezoid cross-section. Anal. Chem..

[27] Kumar D.P., Amgoth T., Annavarapu C.S.R. (2019). Machine learning algorithms for wireless sensor networks: A survey. Inf. Fusion.

[28] Harnsoongnoen S., Wanthong A. (2021). A non-contact planar microwave sensor for detection of high-salinity water containing NaCl, KCl, CaCl_2_, MgCl_2_ and Na_2_CO_3_. Sens. Actuator B Chem..

[29] Guo J.J., Liu X.Q., Sun Z.S., Zheng X.Q., Sung H.K., Yao Z., Li Y., Li Y.Y. (2024). An intelligent dual-sensing e-skin system for pressure and temperature detection using laser-induced graphene and polydimethylsiloxane. Mater. Des..

[30] Harnsoongnoen S., Loutchanwoot P., Srivilai P. (2023). Sensing high 17β-estradiol concentrations using a planar microwave sensor integrated with a microfluidic channel. Biosensors.

[31] Amineh R.K., Ravan M., Tandel D. (2023). Detection of water pollutants with a nonuniform array of microwave sensors. IEEE Trans. Instrum. Meas..

[32] Zhu Z.X., Li S., Wu D., Ren H., Ni C., Wang C.L., Xiang N., Ni Z.H. (2022). High-throughput and label-free enrichment of malignant tumor cells and clusters from pleural and peritoneal effusions using inertial microfluidics. Lab Chip.

[33] Kazemi N., Gholizadeh N., Musilek P. (2022). Selective microwave zeroth-order resonator sensor aided by machine learning. Sensors.

[34] Rao T.N. (2018). Validation of Analytical Methods.

[35] (2014). ICH Harmonized Tripartite Guideline. Validation of Analytical Procedures: Text and Methodology Q2 (R1).

[36] Shrivastava A., Gupta V.B. (2011). Methods for the determination of LOD and LOQ. Chron. Young Sci..

[37] Muñoz-Enano J., Vélez P., Gil M., Martín F. (2020). Planar microwave resonant sensors: A review and recent developments. Appl. Sci..

[38] Wang B., Long J., Teo K.H. (2016). Multi-channel capacitive sensor arrays. Sensors.

