# Supplementary material for: An Integrated Microfluidic Microwave Array Sensor with Machine Learning for Enrichment and Detection of Mixed Biological Solution
Source: Biosensors (Basel). 2025 Jan 13;15(1):45. doi: 10.3390/bios15010045 (PMC11764409; doi:10.3390/bios15010045)
Supplement: Supplementary file 1 [file biosensors-15-00045-s001.zip › biosensors-3384381-supplementary.pdf]

Supplementary Material

An Integrated Microfluidic Microwave Array Sensor with Machine Learning for Enrichment and Detection of Mixed Biological Solution

Sen Yang [1,†], Yanxiong Wang [1,2,†], Yanfeng Jiang [1] and Tian Qiang [1,*]

[1] School of Integrated Circuits, Jiangnan University, Wuxi 214122, China; 6221922014@stu.jiangnan.edu.cn (S.Y.); 7231923001@stu.jiangnan.edu.cn (Y.W.); jiangyf@jiangnan.edu.cn (Y.J.)
[2] School of Internet of Things Engineering, Jiangnan University, Wuxi 214122, China
* Correspondence: qtknight@jiangnan.edu.cn
† These authors contributed equally to this work.

1. Supplementary Note S1: The image of sample Preparation

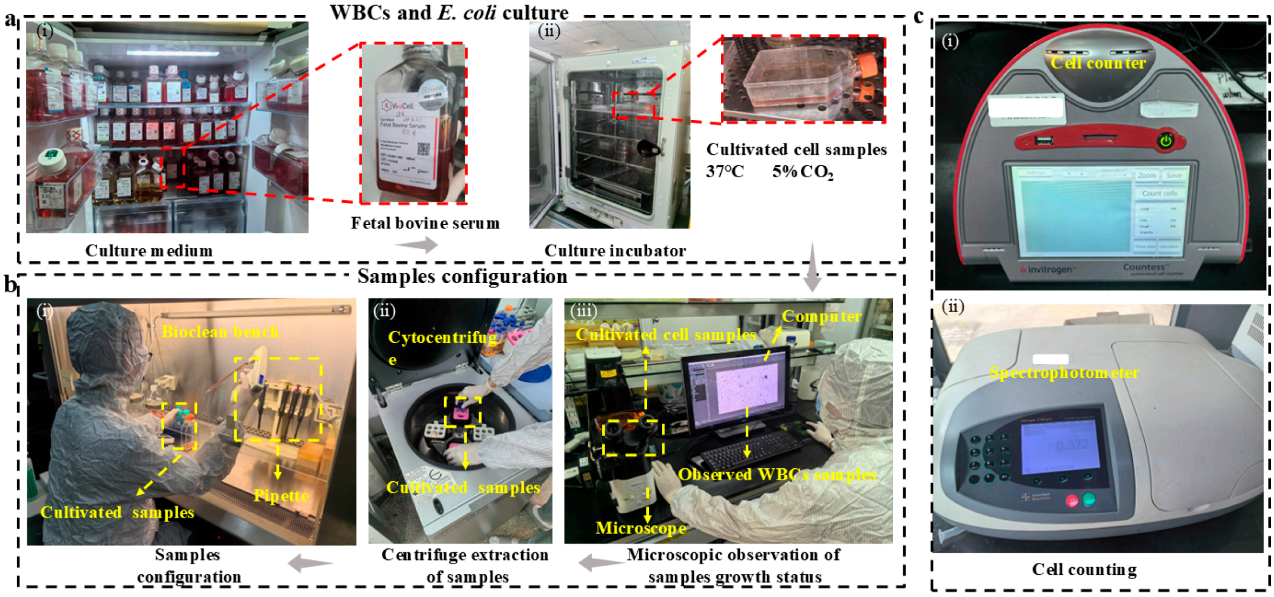

Figure S1. Preparation of WBCs and *E. coli* samples: (a) Culture environment and process, (a-i) Bovine serum stored in the refrigerator, (a-ii) WBCs are cultured in an incubator, (b) Samples preparation process, (b-i) Preparation of mixed samples of WBCs and *E. coli*, (b-ii) Centrifuge to extract the cell sample, (b-iii) Microscopic observation of the WBCs sample, (c) Instruments for counting WBCs and *E. coli*, (c-i) Device for WBCs counting, (c-ii) Spectrophotometer for *E.coli* enumeration.

Figure S1 represents the process of cell sample preparation. The bovine serum is shown in Figure S1(a-i). The cell culture incubator is shown in Figure S1(a-ii). The cell samples are prepared in a sterile workbench, as shown in Figure S1(b). The white blood cells (WBCs) are counted by cell counting plates and a cell counter from Figure S1(c-i), while the optical density (OD) of *E. coli* is measured using a spectrophotometer at 600 nm wavelength, as shown in Figure S1(c-ii).

2. Supplementary Note S2: Calculation table of statistical parameters

Table S1. Statistical analysis of calculation results.

NAME	LOD (10^5/mL)	LOQ (10^5/mL)	CV (%)
Resonator_1	0.331385455	1.104618184	0.033027731
Resonator_2	0.956260065	3.187533551	0.038760873
Resonator_3	0.520026023	1.733420075	0.056577144
Resonator_4	0.140761384	0.469204613	0.029658113
Capacitor_1	0.262298294	0.874327647	2.910869828
Capacitor_2	0.344472317	1.148241058	3.230802181
Capacitor_3	0.237516626	0.791722086	2.411059598
Capacitor_4	0.188038761	0.626795871	1.474870185

Statistical analysis (e.g. coefficient of variation (CV), limit of detection (LOD), and limit of quantification (LOQ) etc.) was performed, the steps and results of the calculations are as follows[1-3]:

$$CV = \frac{\sigma}{\mu} \times 100\%$$

$$LOD = 3.3 \times \frac{SD}{m}$$

$$LOQ = 10 \times \frac{SD}{m}$$

where σ is the standard deviation of the sample, μ is the mean of the sample, SD is the standard deviation of the frequency response, m is the slope of the regression line.

In summary, the microwave resonators and capacitive sensors both perform excellently in terms of sensitivity, detection limits, and stability. Resonant sensors exhibit higher sensitivity and lower measurement variability, which gives them better performance in the detection of low concentration samples. The coefficients of variation are higher for capacitive sensors than for resonant sensors, but are within reasonable limits of experimentally measured errors. However, these differences are reasonable and likely arise from the following causes:

(1) Differences in Sensor Construction and Working Principles: Capacitance sensors typically feature a forked finger structure, which is simple but susceptible to instability due to the influence of various factors on capacitance. In contrast, resonant sensors typically employ a split-ring design, which is more complex and provides more stable measurement results because the resonance frequency is primarily influenced by the physical properties of the measured object[4,5]. Additionally, the sensors operate at different frequencies, the capacitance sensor operates at 1 MHz, while the resonant sensor operates at frequencies above 1.5 GHz.

(2) Sample Characteristics Difference: *E. coli*, as a microorganism, exhibits greater variability in its group characteristics compared to WBCs. At higher concentrations, *E. coli* groups can show more significant changes in physical properties, leading to increased fluctuations in measurement results.

(3) Cleaning Issues: Compared to WBCs, cleaning *E. coli* is relatively more difficult. During multiple measurements, the sensor may not be thoroughly cleaned, leading to some residual material, especially at higher concentrations. These residues could affect the accuracy of subsequent measurements.

References:

[1] T.N. Rao, 2018, Validation of analytical methods; Intech Open: London, ISBN 978-1-78923-085-7.

[2] ICH Harmonized Tripartite Guideline, 1996, Validation of analytical procedures: text and methodology Q2 (R1). In: Proceedings of the International Conference on Harmonization, pp. 11–12.

[3] A. Shrivastava, V. B. Gupta, Methods for the determination of LOD and LOQ. Chron. Young Sci. **2** (2011) 21–25.
https://doi.org/10.4103/2229-5186.79345.

[4] J. Muñoz-Enano, P. Vélez, M. Gil, F. Martín, Planar microwave resonant sensors: a review and recent developments. Appl. Sci. 10 (2020) 2615.
https://doi.org/10.3390/app10072615.

[5] B. Wang, J. Long, K. H. Teo, Multi-channel capacitive sensor arrays. Sensors, 16 (2016) 150.
https://doi.org/10.3390/s16020150
